# The effect of impaction energy and bone quality on cementless femoral implant fixation and femoral periprosthetic fracture risk

**DOI:** 10.1302/2046-3758.157.BJR-2025-0532.R1

**Published:** 2026-07-17

**Authors:** M. Abdulhadi Alagha, Rishabh Gupta, Chloe Jordan, Geoffrey Ng, Justin Cobb

**Affiliations:** 1 MSk Lab, Department of Surgery and Cancer, Faculty of Medicine, Imperial College London, London, UK; 2 Mid and South Essex Trust, Broomfield Hospital, Chelmsford, UK; 3 Plastic Surgery Unit, Addenbrookes Hospital, Cambridge University Hospitals, Cambridge, UK; 4 Medical Biophysics, Western University, London, Canada; 5 Medical Imaging, Western University, London, Canada; 6 Surgery, Western University, London, Canada; 7 Robarts Research Institute, Western University, London, Canada

**Keywords:** Digital image correlation, Impaction, Cementless fixation, periprosthetic fractures, implant fixation, strains, osteoporotic bones, femoral implants, synthetic bone models, Sawbones, soft-tissue, bone quality, bone models

## Abstract

**Aims:**

Cementless fixation is the predominant method used in hip arthroplasty worldwide; however, seating implants via impaction remains a balancing act – too much energy may cause fracture, and too little may not seat the implant adequately. Currently, the optimal energy level required for femoral implant fixation is unknown, which adversely affects the variability of long-term implant stability. This study investigated how varying impaction energies affect strain behaviour, fracture risk, and implant fixation in synthetic bone models using cementless femoral implants.

**Methods:**

A standardized surgical technique was used to prepare a total of 20 synthetic bone models (Sawbones) – 12 healthy and eight osteoporotic – for femoral impaction. The implants were impacted at three different energy levels (low, medium, and high) using a validated in vitro rig simulator for soft-tissue compliance, with four specimens tested at each velocity and bone type. Strain behaviour and fracture risk, measured as peak strain, were analyzed using digital image correlation with two high-speed cameras. Implant stability was assessed via pull-out fixation tests.

**Results:**

Higher impaction energy increased fracture risk but did not significantly increase implant stability. In healthy bone models, mean tensile peak strain was 0.31, 0.69, and 0.97 for low, medium, and high energy strikes, respectively. Osteoporotic models showed the greatest difference between the low and medium energies during the initial two strikes, with peak strains of 0.42 to 0.46 versus 0.79 to 0.84, with subsequent strikes resulting in minimal variation between the two velocities. Implant pull-out forces were similar among the different impaction energy levels within each group (healthy p = 0.105, osteoporotic p = 0.686), and consistently lower in osteoporotic bones across all energy levels.

**Conclusion:**

These biomechanical findings on synthetic bones confirm that multiple lower-energy strikes lead to similar implant fixation outcomes with lower risk of periprosthetic fracture (lower peak strain).

Cite this article: *Bone Joint Res* 2026;15(7):892–902.

## Article focus

- To investigate the biomechanical effects of varying impaction energy levels during cementless femoral implant insertion.
- To assess strain behaviour, fracture risk, and implant fixation in synthetic models simulating both healthy and osteoporotic bone conditions.

## Key messages

- Higher impaction energy increases the risk of periprosthetic fracture but does not significantly enhance implant stability.
- Digital image correlation revealed that peak strain increases with impaction velocity. However, pull-out fixation strength did not show significant improvement with higher-energy strikes.
- Multiple lower-energy strikes provide a safer alternative with comparable biomechanical fixation outcomes.

## Strengths and limitations

- Use of a validated in vitro testing rig simulating soft-tissue compliance.
- Application of high-resolution digital image correlation for precise strain measurement.
- Inclusion of both normal and osteoporotic synthetic bone models, increasing relevance to diverse patient populations.
- The use of synthetic bone models, while enabling controlled biomechanical testing for varying bone mineral density, limits the study’s generalizability due to their inability to fully replicate the anatomical complexity and biological variability of human bone, and the findings do not account for long-term clinical outcomes or in vivo healing responses.

## Introduction

Total hip arthroplasties (THAs) are effective orthopaedic surgeries, typically indicated in patients with end-stage osteoarthritis.^1^ Cementless fixation for the femoral stem became popular in the late 1980s and is the fixation of choice in most developed countries, including the USA,^2^ Germany,^3^ Japan,^4^ Australia,^5^ England and Wales, Canada, and Italy.^6^ However, there remains controversy about the use of cementless fixation in osteoporotic bones, as it has a higher risk of failure in this patient group.^7^

Initial cementless fixation relies on sequential impaction strikes to provide press-fit stability. Long-term stability is provided by bone ingrowth, achieved via good initial bone and implant apposition.^8^ Appropriate impaction technique involves two key stages: a broaching step to prepare the intramedullary canal and a femoral implant insertion step, both of which are crucial to ensure satisfactory long-term osseointegration occurs between the cementless implant and the bone.^9^ The impaction energy must be sufficient to seat the broach/implant fully but not so excessive as to result in fractures.^10^ Previous studies investigated the effect of strike technique on the seating and stability of the cementless acetabular component, assembling femoral head and influencing taper-trunnion stability.^11-13^

Currently, surgeons rely on proprioceptive estimations of ideal impaction energy and number of strikes to achieve an optimal balance between stem/broach seating and fracture risk.^14^ An understanding of the biomechanical properties of a human femur may provide empirical evidence to improve fixation and minimize fracture risk. Strain measurement techniques can reliably predict regions of high stress in the bone during impaction. Cummins et al^15^ calculated the threshold force required for femoral impaction grafting using strain gauges, which was further validated by Flannery et al.^16^ Strain gauges, however, only allow for discrete point measurements to be obtained,^17^ making it challenging to measure strain over the full duration of impaction. Non-contact optical digital image correlation (DIC) can give detailed directional strain magnitudes on 3D surfaces and is suitable for complex irregular structures such as femoral bone models.^18,19^ DIC captures sequential images of a specimen during mechanical testing and computes deformation, strain, and displacement through analysis of spatial correspondences.^20,21^ Previous studies used DIC to measure failure in proximal femora under quasistatic loading conditions.^22,23^ If applied to the dynamic impaction process, DIC could yield new understandings of how strain fields are generated.

The aim of this study is twofold: firstly, we propose a new method combining DIC with high-speed cameras to capture the rapid changes in bone strain during femoral impaction; secondly, we investigate strain behaviour, fracture risk, and implant fixation in synthetic bone models during cementless femoral broaching and stem impaction. In other words, do higher impaction strikes lead to higher fracture risks or better fixation outcomes? We hypothesized that higher impaction forces would increase bone strain, potentially raising fracture risk while also improving implant fixation.

## Methods

### Specimen preparation

In this study, a total of 20 synthetic bones were used: 12 healthy and eight osteoporotic. All Sawbones models were adult left femora and manufactured by Sawbones (Pacific Laboratories, Sweden; models 1130 and 1130-130). The healthy and osteoporotic specimens were composed from 30 pounds per cubic foot (PCF) and 15 PCF respectively, thereby representing normal and low bone density. All specimens, healthy and osteoporotic, were prepared using a standard THA direct anterior approach.^24^ Instruments for cementless THA (Furlong Evolution; JRI Orthopaedics, UK) were used to prepare femora for impaction and during impaction testing itself. Four specimens were tested at each impaction velocity and bony type.

Using an oscillating saw, each specimen was transversely cut at 17 cm proximal to the intercondylar fossa. The mid-point between the lesser trochanter and the femoral head was identified using a resection guide. The neck was then resected at 45° to the long axis of the femur. To gain access to the medullary canal, a box osteotome was used to remove a section of cancellous bone from the upper-lateral aspect of the femoral neck.^24^

To open the medullary canal, a T-handled taper reamer was used to prepare the canal for broaching, with care taken to ensure that the reamer was aligned with the femoral shaft axis. The reamer was used until the minimum depth, as indicated on the instrument, had been met.

To prepare the intramedullary canal for receiving the femoral stem implant, the smallest-size broach (size 6) was attached to the broach handle and repeatedly hammered in and out to remove the synthetic cancellous bone. The broach was impacted until the proximal part of the broach handle was aligned with the osteotomy level. To avoid varus positioning, a posterior lateral pressure was applied to the broach handle during impaction.

### Application of the speckle pattern

For the DIC system to accurately capture data, a specially prepared high-contrast speckle pattern is applied to the surface of interest.^25^ DIC records and tracks the movement of the speckle pattern during mechanical testing, thus allowing surface strain to be calculated.^26^ To achieve the speckle pattern, white and black paints were applied to each specimen two hours prior to impaction. The anterior surface of the femur was sprayed with a coat of white paint (VHT SP101; Sherwin-Williams, USA) and set aside to dry for 20 minutes.^27^ Black spray paint (VHT SP102) was then used to apply the speckles to achieve the required pattern. All specimens were sprayed from a distance to ensure only thin layers of paint were applied. GOM Correlate Professional (GOM UK, UK), a post-processing software, was used to assess pattern quality prior to testing.^22^

### Setup

**Fig. 1:**
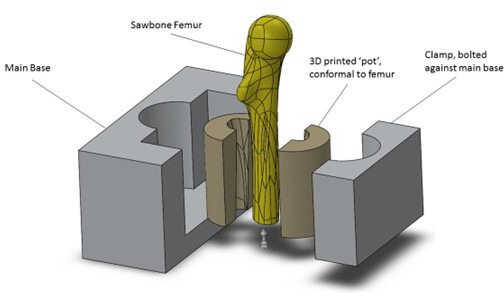
Assembly of platen and bone onto the rig.

When a specimen was ready for testing, it was placed into a custom 3D-printed holder and secured onto the impaction platen (Figure 1) in a vertical loading axis at a 7° tilt.^28^

**Fig. 2:**
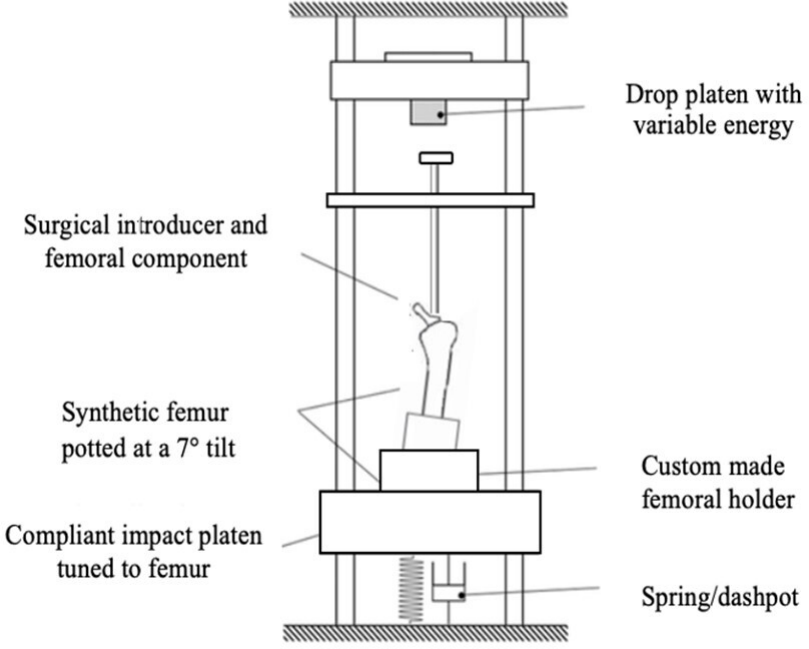
Experimental setup illustrating the in vitro drop rig used for impaction.

As seen in Figure 2, the impaction platen is secured to a spring/dashpot support. This provides the same displacement and acceleration rates as would be observed intraoperatively, thus accounting for soft-tissue compliance and movement of the human femur.^29^ Strikes were delivered to each specimen by releasing the drop platen onto a secured broach handle below and subsequently the specimen. The rig drop platen was used to deliver impaction strikes to the specimen. A platen drop weight of 1.2 kg was chosen, representing the average mass of a THA surgical mallet. To vary the energy at which the specimens were impacted, the platen was released from predetermined heights corresponding to specific energies.

### Testing

Using the in vitro drop rig (Figure 2), femoral broaches of increasing size (sizes 7, 8, 9, and 10) were impacted into the femoral canal of each specimen. Beginning with a size 7 rasp, consecutive strikes were applied to the broach handle by releasing the drop platen to repeatedly impact the rasp into the femur. Consecutive strikes were applied until the rasp was seated. The interval between individual strikes was not standardized or recorded, owing to pragmatic considerations and the requirement for periodic recalibration of the measurement apparatus. Broaches were hammered out of the femoral canal following every third strike. After withdrawal, the broach was cleared of fragments and a further three strikes were then applied, with this process continued until the broach was seated.

The broach was considered seated once the cutting teeth were parallel to the depth of the neck resection, at which point the broach was hammered out, removed from the handle, and replaced with the next size, and the impaction process was restarted. The broaching process was repeated, sequentially increasing the broach size until size 10, which was the size of the selected implant.

The intramedullary canal was deemed prepared for receiving the cementless stem prosthesis when the final broach, size 10, had been adequately seated and disengaged from the canal. A size 10 JRI Furlong femoral stem implant was then attached to an introducer and engaged into the femoral canal by hand. The introduction of the stem was completed by impacting the introducer until a tight metaphyseal fit was achieved.^24^

Impaction energy levels were divided into three categories: low, medium, and high. Each energy level was defined based on measurements recorded in a study by Doyle et al.^30^ In that study, the surgeon simulated three levels of impact to the cadaveric specimens for both acetabular and femoral components: one mimicking the force used for patients with healthy bones, another mimicking the gentleness employed for patients with osteoporotic bones, and an intermediate impact. Three energy levels were recorded: E_Low_ = 1.26 J; E_Medium_ = 4.84 J; and E_High_ = 10.78 J.

Given that energy (J) is the product of mass (platen drop, kg) and the square of the velocity, $E(J)=\frac{1}{2}m(kg)v^{2}$, energy levels generated for our study used a standard ‘mallet’ weight of 1.20 kg and varied velocities (Table I). The maximum velocity achieved by the rig was 4.08 m/s; therefore, the high energy level in our study was 9.99 J. On the healthy synthetic bone specimens, this protocol was performed at low, medium, and high impaction energies, while for the osteoporotic specimens the protocol was only performed at low and medium energy levels since high velocity fractured low bone mineral density (BMD) Sawbones during our pilot testing.

**Table I.** The impaction energy levels investigated: low, medium, and high.

Energy level	Low	Medium	High*
Drop mass, kg	1.20	1.20	1.20
Drop velocity, m/s	1.45	2.84	4.08
Energy, J	1.26	4.84	9.99

* Only healthy bones were impacted at this energy level.

In all, 20 synthetic bones were tested, totalling 600 impaction strikes; 12 normal BMD specimens were impacted at low, medium, and high energy levels, and eight low BMD samples were impacted at low and medium energy levels (n = 4 per energy level). As a result of the large number of strikes required to seat both broaches and implants at low and medium velocities in healthy bones, data analysis occurred for every third strike (i.e. strike 3, strike 6, strike 9…), and the average peak strain was calculated across velocities. Subsampling repeated loading events is common in multicycle DIC studies, where representative or every nth cycle is analyzed once strain patterns are shown to be stable. Preliminary inspection of early, mid, and late strikes in our dataset confirmed no systematic drift in strain distribution. Accordingly, analyzing every third strike provided reliable strain characterization while reducing noise and computational burden. For high velocity in healthy bones and for low and medium velocities in osteoporotic bones, data analysis occurred for each strike.

### Digital image correlation

**Fig. 3:**
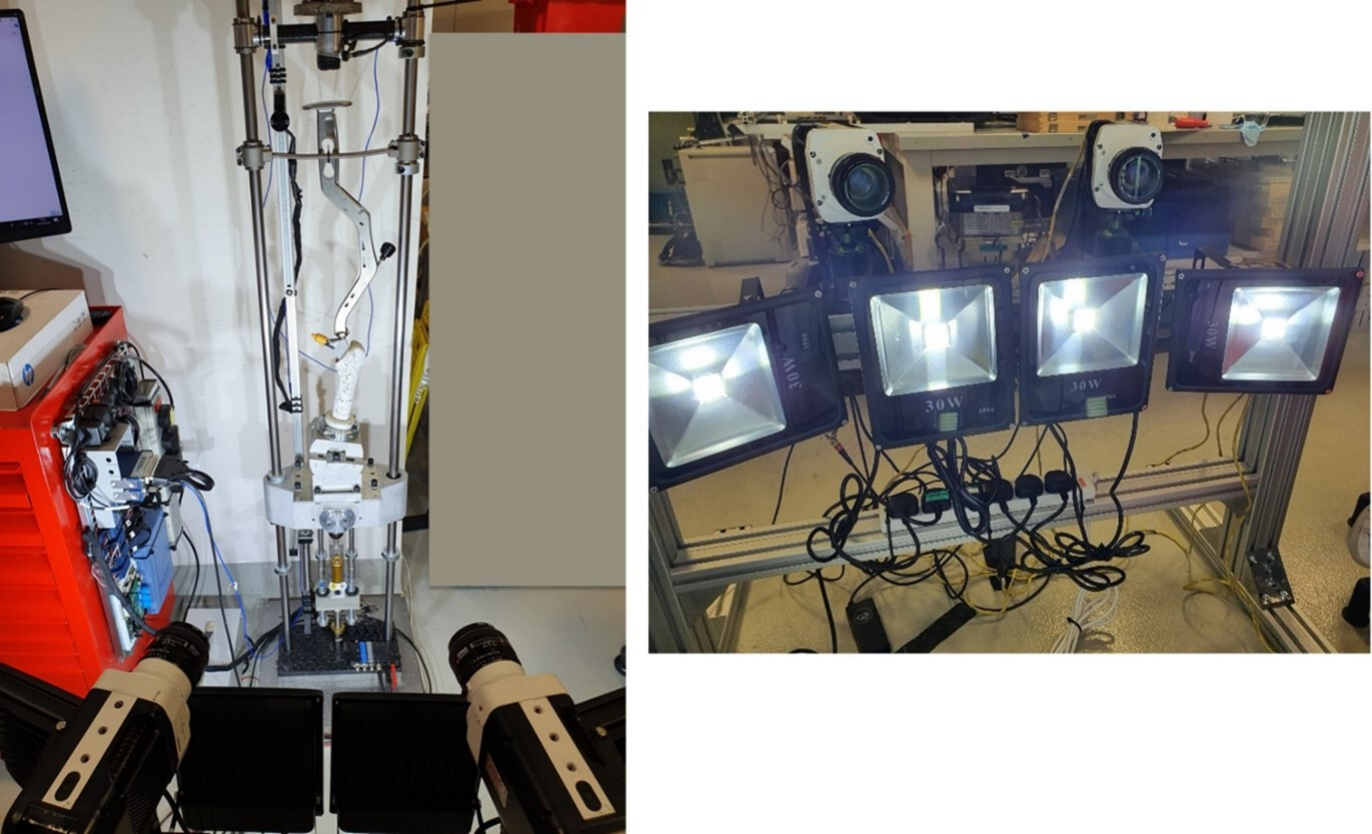
The 3D digital image correlation (DIC) system setup. Left: a photograph of the 3D DIC setup; right: a photograph of the cameras and light setup.

To measure strain during mechanical testing, images were captured using a 3D DIC system.^23,31^ Two high-speed cameras (Phantom Miro, Vision Research Inc., USA) were set up as shown in Figure 3.^32,33^ Both cameras were secured to a custom-made stand and rotated internally at a 12.5° angle. The impaction process was captured at 3,268 frames per second at a resolution of 768 × 768 pixels. The DIC system was calibrated prior to testing using a calibration model to establish the event volume. To increase specimen visibility during testing, additional lighting was used to illuminate the synthetic surface pattern.^34^ For the osteoporotic bones, each strike was analyzed for peak strain measurements in regions representing Gruen zones (GZs) 1, 2, 6, and 7. On the 12 healthy bones, the peak strain measurements were measured at regions representing GZs 1, 2, 3, 5, 6, and 7.

DIC measures deformation by tracking changes in the surface pattern of the material (bone) between a reference (prestrike) state and the deformed state. The strain, therefore, is calculated as the change in length per unit length, relative to that reference configuration from the previous strike. The DIC system quantified surface strain in the direction of highest deformation, corresponding to the local maximum principal strain. The direction of deformation was qualitatively consistent across specimens and energy levels. Peak strain values represent the mean of maximum strain magnitudes measured within predefined regions of interest. Peak strain was extracted at each strike within each region of interest. For each specimen, the mean peak strain was then calculated across all analyzed strikes. Group-level values represent the average of these specimen-level means.

### Fixation strength

**Fig. 4:**
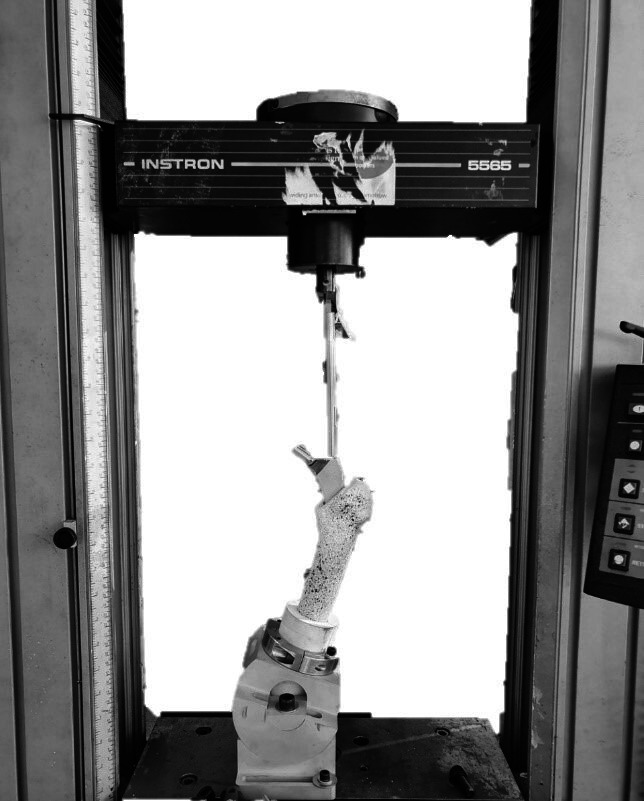
Pull-out test setup using a uniaxial Instron.

To evaluate the initial stability of the femoral stem implant, pull-out fixation tests were conducted on a uniaxial loading testing machine (Instron model 5565; Instron, UK) (Figure 4).^35^ The femoral holder was secured to the test frame so that the actuator motion of the Instron was perpendicular to the axis of the femur. Implants were pulled out using an 11 mm diameter steel rod which fixed distally to the implant and proximally to the machine actuator head. Tensile load was applied at a crosshead displacement rate of 0.2 mm/min until the implant dislodged from the specimen.^36^

### Statistical analysis

Variations in strain data between low-, medium-, and high-velocity strikes in normal and low BMD specimens were evaluated for statistical differences. Data were assessed for normality assumptions using the Shapiro-Wilk test and visual inspection of box plots. A one-way analysis of variance (ANOVA) determined statistical significance for normally distributed data, with post-hoc pairwise independent-samples *t*-tests where significant effects were observed, while for non-normally distributed data the Kruskal-Wallis test with post-hoc Mann-Whitney U test and Bonferroni correction were applied. The level of significance was established as *α* = 0.05 for all tests. All analyses were performed using SPSS v. 26.0 (IBM, USA) and Microsoft Excel Version 22.0 (Microsoft, USA).

## Results

**Fig. 5:**
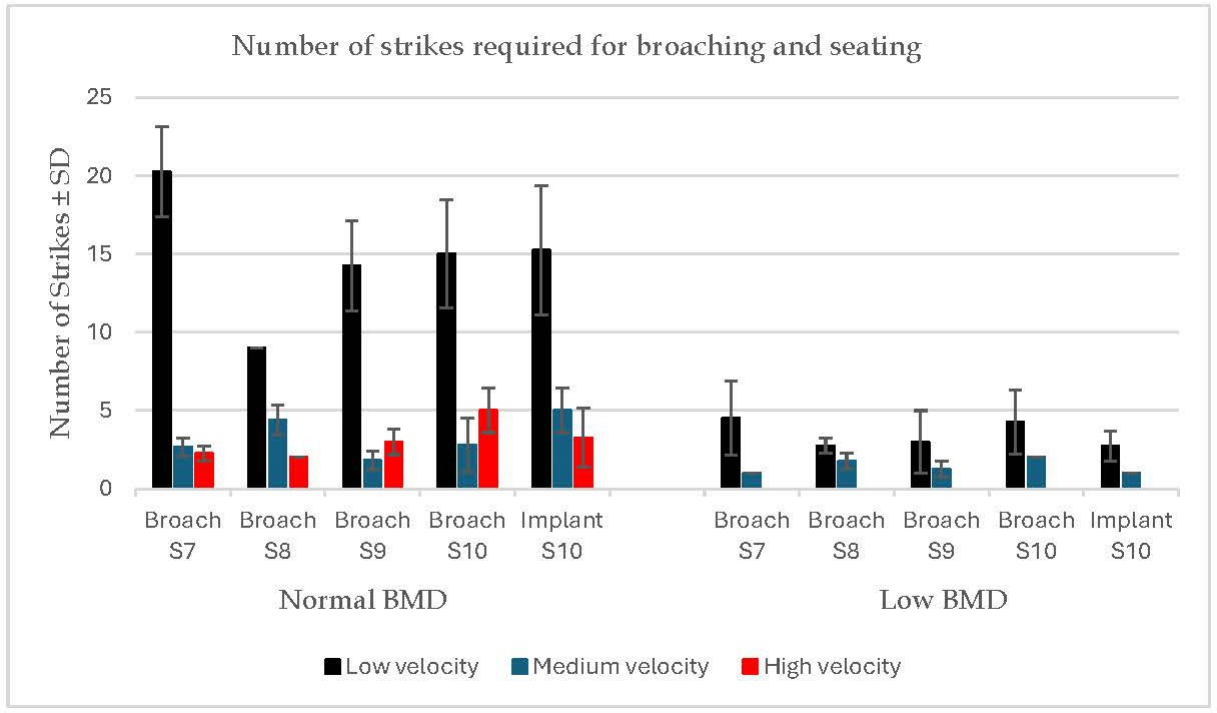
Mean number of strikes with SD required to seat broaches 7 to 10 and a size 10 implant at different mallet velocities in both normal bone mineral density (BMD) (healthy) and low BMD (osteoporotic) bones.

In normal BMD bone using low velocity strikes, the mean cumulative number of broach impactions required was 58 (SD 5.3), while only ten cumulative broach impactions were needed using either medium (SD 2.1) or higher (SD 1.7) velocity. In low BMD bone, 14 (SD 3.7) cumulative broach impactions were needed using low velocity compared with seven (SD 0.7) cumulative strikes using medium velocity. For implantation in normal BMD bone, a mean of 15 cumulative strikes (SD 4.1) were needed using low velocity compared with five (SD 1.4) using medium velocity and three (SD 1.9) using high velocity. In low BMD bone, three cumulative impactions (SD 0.9) were needed to seat the implant, while just one medium-velocity impaction was sufficient. In both bone types, these differences were highly significant for both broaching (p = 0.001, p = 0.043, two-way ANOVA) and seating the implant (p < 0.001, p = 0.013, two-way ANOVA), respectively. For normal BMD, no difference was detected regarding the number of strikes between medium and high velocities for broaching (p = 0.873, Welch independent-samples *t*-test) and implant seating (p = 0.193, Welch independent-samples *t*-test; p = 0.561, Sidak’s adjusted). Figure 5 shows the mean number of strikes during mechanical testing for both specimen types at each impaction velocity.

**Fig. 6:**
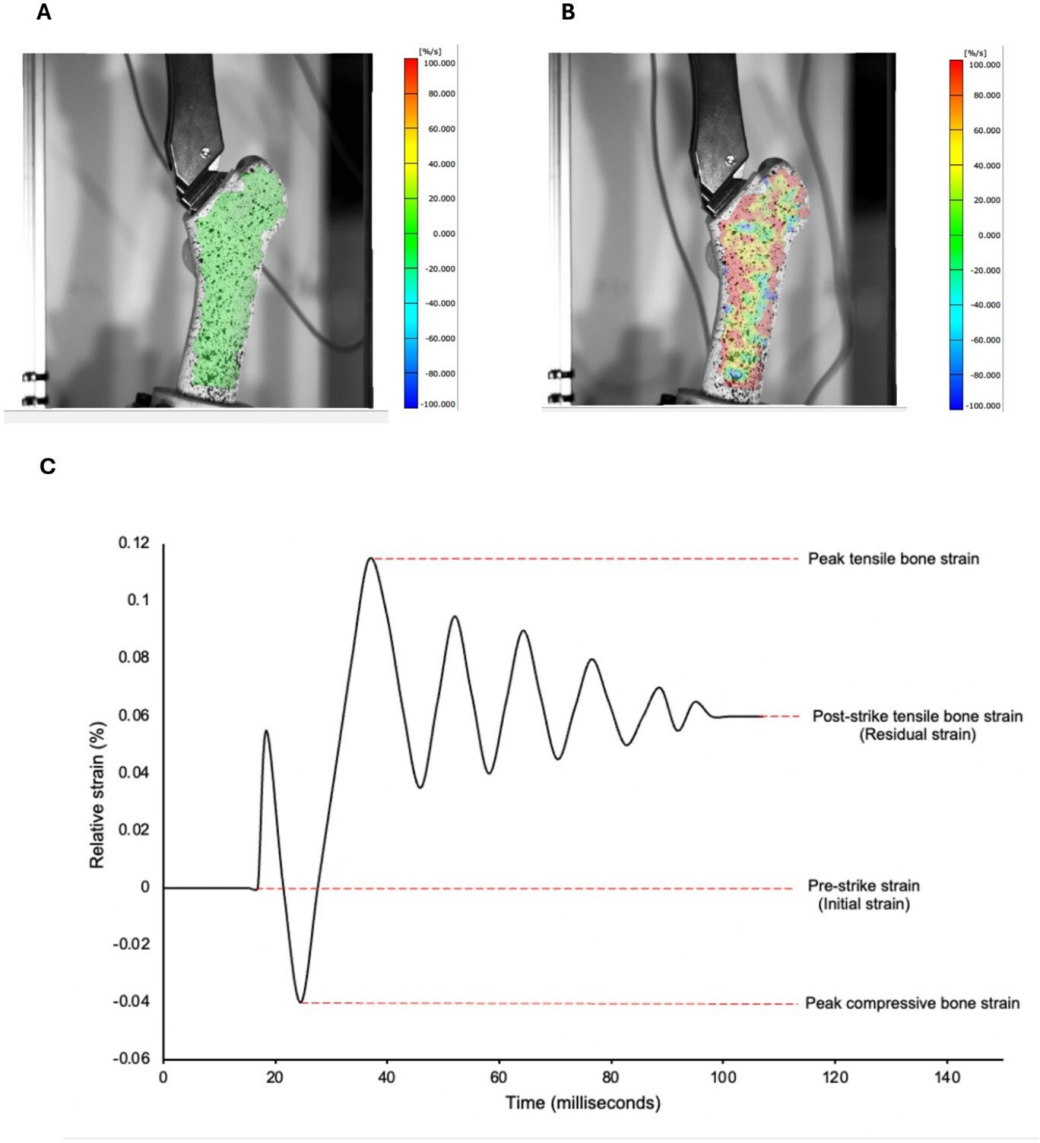
a) Illustrative contour plot before impaction. b) Illustrative contour plot during broaching impaction. c) Strain behaviour during impaction.

Figure 6a represents a contour plot of DIC strain for a normal BMD specimen before impaction. Figure 6b represents a contour plot of DIC strain for a normal BMD specimen undergoing implant insertion with high velocity. The pattern of strain is shown on the anterior proximal femur, and the contour plot goes from −100%/s to +100%/s. Figure 6c is representative of a general pattern of strain observed during impaction in bone samples across all impaction velocities in GZs 1, 2, 6, and 7 in the direction of highest deformation. As shown in Figure 6c, after the strike an initial peak compressive strain was followed by a tensile strain. This was further followed by a post-strike tensile strain which diminished in magnitude prior to stabilizing.

**Fig. 7:**
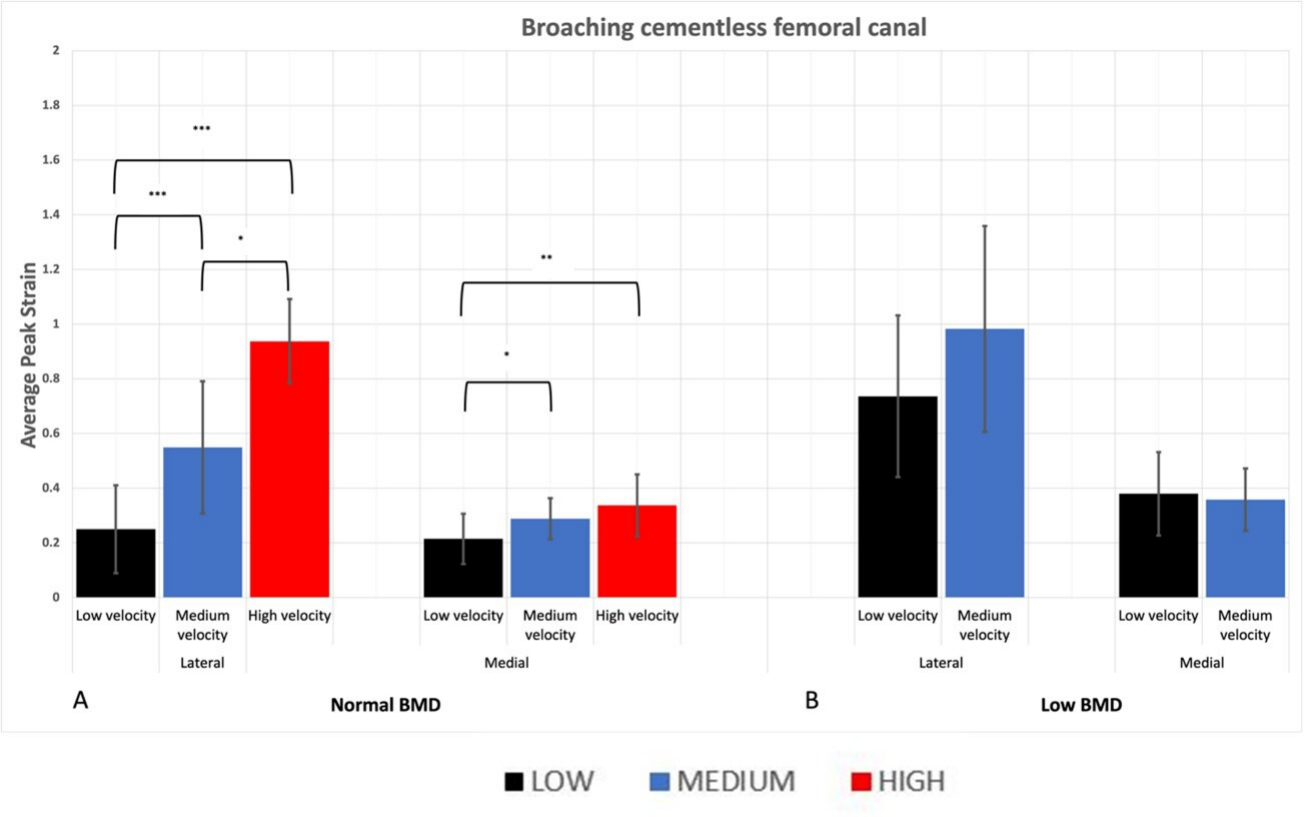
Mean peak strain during broaching of a cementless femoral canal. a) Normal bones. b) Low bone mineral density (BMD) bones. Plotted group means reflect the average of specimen-level mean peak strains, where each specimen’s value was calculated by averaging peak strain across all analyzed strikes within each predefined region of interest. Data represent peak strain at each Gruen zone during impaction. In all, 20 synthetic femora were tested, comprising 12 normal BMD specimens (impacted at low, medium, and high velocities) and eight low BMD specimens (impacted at low and medium velocities; n = 4 per energy level). Low and medium velocity, normal BMD: due to the high number of strikes required to seat broaches and implants, analysis was performed on every third strike (i.e. strike 3, 6, 9…), with mean peak strain calculated across these strikes. High velocity, normal BMD, and all strikes for osteoporotic bones: data were analyzed for each strike. Values plotted represent the average peak strain across all analyzed strikes per implant. Error bars indicate SD across bones. Statistical significance: *p < 0.05; **p < 0.01; ***p < 0.001, independent-samples *t*-test assuming equal variance.

For normal BMD specimens, mean tensile peak strain was 0.31, 0.69, and 0.97 for low-, medium-, and high-energy strikes, respectively. The average peak strain increased with each impaction velocity in the lateral GZs (low vs medium p < 0.001; medium vs high p < 0.05; and low vs high p < 0.001) (Figure 7a). For the medial GZs, the average peak strain increased for medium velocity compared with low velocity (low vs medium p < 0.05, independent-samples t-test), but did not increase further for high impaction velocity (Figure 7a). Osteoporotic models showed the greatest difference between the low and medium energies during the initial two strikes, with peak strains of 0.42 to 0.46 versus 0.79 to 0.84, with subsequent strikes resulting in minimal variation between the two velocities. In low BMD samples, there was no difference in peak strain for the different velocities in either the lateral side (p = 0.080, between group one-way ANOVA) or medial side (p = 0.833, between group one-way ANOVA) (Figure 7b).

**Fig. 8:**
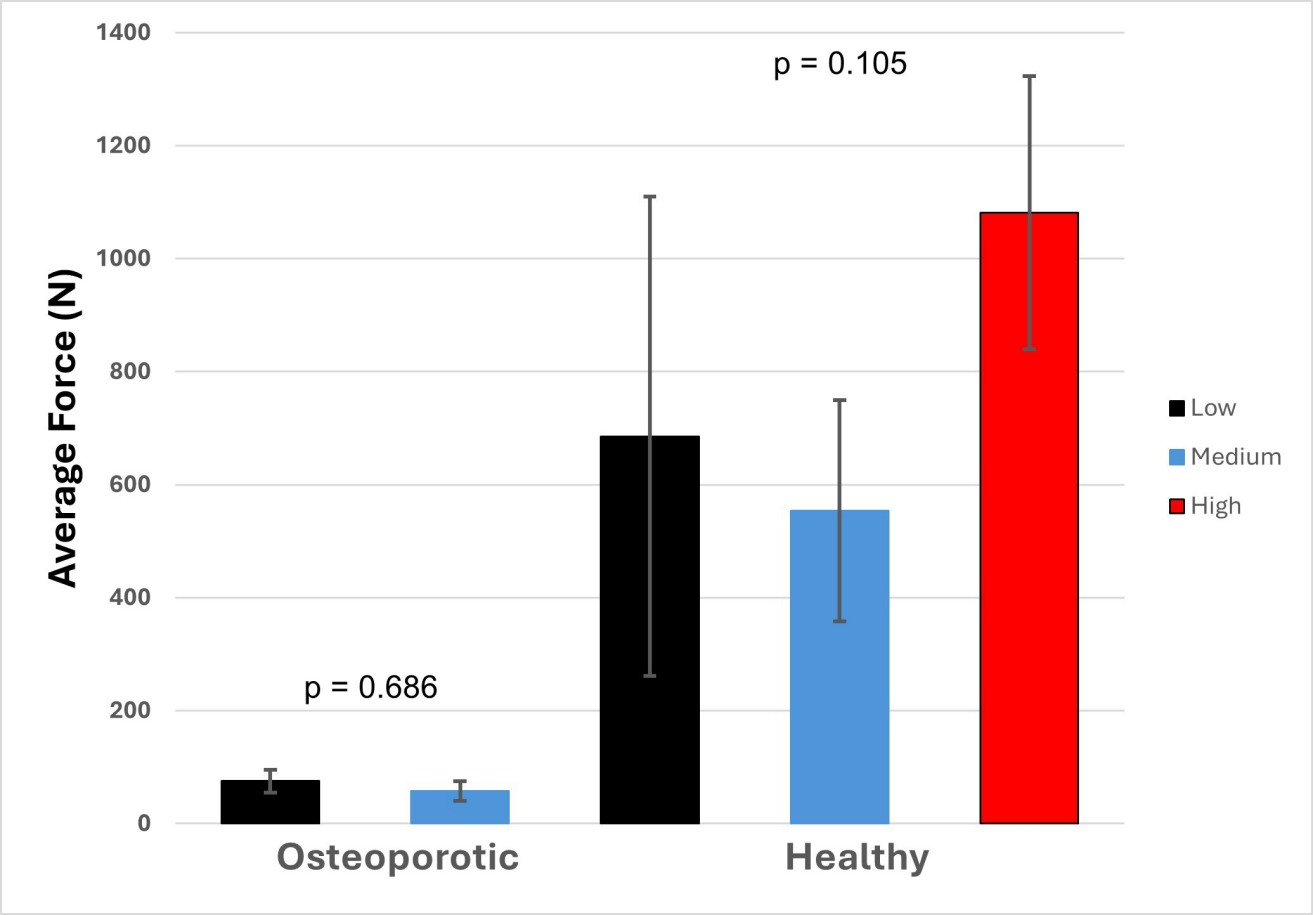
The mean force required to remove the implant from osteoporotic and healthy femoral Sawbones following impaction at different energy levels, with 95% CIs. Statistical significance: *p < 0.05; **p < 0.01; ***p < 0.001. Osteoporosis (p = 0.686, Kruskal–Wallis test); healthy (p = 0.105, Kruskal–Wallis test between groups).

The force required to remove specimens impacted by low or medium velocity strikes in normal bone showed a mean of 685 N (269 to 1,100) and 553 N (362 to 746), respectively. Statistical testing did not detect a difference between these groups (p = 0.886, Mann-Whitney U test; p > 0.999 after Bonferroni correction), but the wide ranges indicate considerable uncertainty. A similar pattern of high variability and no statistical significance was observed in osteoporotic bone between low and medium velocities (p = 0.686, Mann-Whitney U test) (Figure 8). The Kruskal–Wallis test showed no overall significant difference between the three groups in healthy bones (H2 = 4.50, p = 0.105). Over 1,000 N (844 to 1,318) force was required to remove specimens impacted by high velocity strikes in normal bone (mean 1,091 N). This represented a roughly 30% increase compared with low velocity, although the difference did not reach statistical significance (p = 0.343, Mann-Whitney U test; p > 0.999 after Bonferroni correction). Similarly, medium velocity strikes required 50% lower removal force than high velocity, with the comparison again not reaching statistical significance (p = 0.029, Mann-Whitney U test; p = 0.086 after Bonferroni correction). There was a seven-fold reduction in the force needed to extract the implant from low BMD specimens.

## Discussion

The most important finding was that increased impaction velocity led to higher levels of peak strain (fracture risk) and did not affect implant stability. In other words, the higher the impaction energy, the higher the resultant strain and the greater the risk of periprosthetic fracture. In terms of implant stability, the 30% difference observed between high and lower velocity impactions in normal bone failed to reach statistical significance owing to the wide variation between specimens. At low BMD, there was no statistically significant difference between the two velocities, meaning that stability remains the same regardless of the amount of force. To our knowledge, this is the first biomechanical study to use DIC technology to analyze strain in synthetic femora in relation to impaction technique for THA.

The reason for this phenomenon may be explained by works by Doyle et al^26^ and Mold et al,^37^ who both highlighted a theory in their works known as the ‘law of diminishing returns’, whereby increased impaction velocity fails to result in increased implant stability and a limit is reached beyond which any additional energy produces diminishing additional fixation. This is highlighted with the higher impaction that did not result in improved implant stability as represented by pull-out force (Figure 8).

For both specimen types, a strain behaviour pattern was identified in the femur during broaching. During impaction, a compressive peak strain was subsequently followed by a tensile peak strain (Figure 6). Both mean peak strain and mean residual strains across all GZs were tensile in the direction of highest deformation. Following the peak tensile bone strain, a period of oscillation occurred which decayed and resulted in post-strike tensile strain. Despite no fractures occurring during mechanical testing using the lower-energy strikes in the osteoporotic bone, both peak and tensile compressive strains are potential contributors to femoral fractures. A similar pattern of strain has been highlighted in a previous study,^26^ in which peak tensile strain, peak compressive strain, and a similar period of oscillation resulting in a post-strike strain in synthetic acetabular components can be identified.

In the healthy and osteoporotic bone specimens, the highest tensile strain levels – peak and residual – were greatest on the lateral aspect of the femur (GZ 1 to 3) in the direction of highest deformation, for all impaction velocities. Previous studies have demonstrated that the risk of fracture is increased when bones are in a tensile state, as a result of weakening of the cortical bone, indicating that the higher the tensile strain, the greater the fracture risk.^17,38-40^ Thus, applying our results with those in the literature, it could be suggested that if fractures had occurred in this study, they would most likely have occurred along the lateral femoral axis, equivalent to GZ 1 to 3. Mirza et al^41^ previously hypothesized that the fracture pattern could be ascribed to the configuration of both the femoral rasp and the femur thus initiating implant–bone contact stresses. Furthermore, a study by Cummins et al^15^ found that although not reaching statistical significance, there was a correlation between the GZ where the largest strain was recorded and the location of the fracture. This was further supported by Flannery et al:^16^ despite most of the fractures occurring on the medial zone of the proximal femur, their study found that the location of maximal strain recorded was closest to that of the fracture, with the number of specimens recording maximal strain at failure in lateral zones being half the number of those in the medial zones (n = 10/n = 19). Multiple factors could account for the variation in the location of maximum strain. In their study, the researchers employed a model that used femoral morphology of revision hip arthroplasty: they used long-stem Exeter implants (Stryker, USA) cemented into femora fixed in cement and applied an Instron machine of increasing force.^16^ On the other hand, in our study, we used synthetic bone models to represent both healthy and osteoporotic bone conditions. These models were prepared and broached for primary short-stem cementless femoral implants using a validated rig that accounts for soft-tissue compliance.

This work should be considered in the context of several limitations. First of all, synthetic bone replicates similar mechanical properties and minimizes the variability among specimens.^42,43^ However, the differences in physical properties are likely to result in differences in the mechanical response during impaction, with the artificial bone geometry only offering an estimation of the complex pelvic anatomy and in vivo operative fixation. Future cadaveric studies are needed to validate our preliminary findings. Furthermore, the number and location of surface points used for the DIC and the accompanying analysis software varied among the samples and were influenced by several variables. These factors included the limited coverage of surface component, as only two cameras were used for capturing images, and the influence of bone curvature. DIC strains were measured relative to a prestrike baseline, and cumulative strain over successive impacts was not assessed. Similarly, global rig oscillation during impaction was not corrected for in the DIC analysis, and therefore measured surface strains may include contributions from overall rig movement rather than purely local bone deformation. Further studies focusing on fatigue or residual strain as well as alternative correction methods may better capture true internal strain redistribution. The use of multicamera stereo DIC technology presents a promising opportunity to capture 3D views of specimens.^44^ Likewise, strain measurements in our study targeted the proximal femur to just below the femoral stem. There is a risk that this might not take into account strain behaviour related to Vancouver type C fractures, which represent approximately a quarter of femoral periprosthetic fractures in cementless THA.^45^ Our impaction protocol employed a vertical loading axis with a 7° tilt. While this approach yielded a standard testing environment across all specimens, it is important to acknowledge that altering the inclination angle may engender a spectrum of strain behaviours and fracture patterns, and thus future studies ought to investigate the effect of the loading angle on bone strain behaviour. Lastly, while our validated impaction rig provides a controlled and reliable method for research purposes, it is important to consider that surgical impaction in operative procedures tends to be less precise and accurate, which may potentially impact the significance and applicability of our data in practical settings.

Primary implant fixation is a critical factor in determining the technical success of the arthroplasty and patient morbidity. Based on this study, multiple lower-impaction strikes may be preferable for the cementless technique to minimize fracture risk and maximize implant stability.

In conclusion, the results of this study may suggest that in both normal- and low-density bones, a high number of low-energy strikes during femoral broaching and implant seating may provide lower peak strains with similar or increased implant stability. The results offer practical guidance for both the assertive and cautious surgeon to handle bone thoughtfully. They also provide experimental support for the continuing adoption of technologies which are able to deliver lower-energy strikes on the grounds that they will likely reduce the risk of fracture without significantly reducing implant stability.

## Data Availability

The data that support the findings for this study are available to other researchers from the corresponding author upon reasonable request.
